# Thermoplastic Overmolding onto Injection-Molded and In Situ Polymerization-Based Polyamides

**DOI:** 10.3390/ma11112140

**Published:** 2018-10-30

**Authors:** Róbert Boros, Praveen Kannan Rajamani, József Gábor Kovács

**Affiliations:** Department of Polymer Engineering, Budapest University of Technology and Economics, 1111 Budapest, Hungary; borosr@pt.bme.hu (R.B.); rajamani@pt.bme.hu (P.K.R.)

**Keywords:** overmolding, in situ polymerization, polyamide

## Abstract

We investigated products manufactured by in situ polymerization, which were reinforced with overmolded ribs. We developed our own mold and prototype product for the project. We used three different materials as preform: a material with a magnesium catalyst, manufactured by in situ polymerization, a Brüggemann AP-NYLON-based in situ polymerization material and an injection-molded PA6 (Durethan B30S, Lanxess GmbH) material. The ribs were formed from the same PA6 material (Durethan B30S, Lanxess GmbH). We examined the effect of the different technological parameters through the pull-off of the overmolded ribs. We measured the effect of melt temperature, holding pressure and holding time, and mold temperature. Considering the individual preforms, we pointed out that monomer migration and binding strength are related, which we concluded from the temperature-dependent mass loss of the materials, measured by thermogravimetric analysis (TGA). Finally, we designed a mold suitable for manufacturing overmolded parts. We designed and built pressure and temperature sensors into the mold to examine and analyze pressures and temperatures around the welding zone of the materials.

## 1. Introduction

The importance of polyamides cannot be questioned as many automotive parts are made from them, among others. Their mechanical properties, such as long-term properties, are increasingly important [1,2]. Also, special technologies are developed which help shape the final parts. Among others, overmolding, special joining techniques [3] and also the development of some special electrical or thermal properties [4] could play a key role in their future development.

Injection molding—today’s most widely used plastic forming technology—is gaining popularity fast in the automotive industry, too. In addition to conventional technologies, increasingly special technologies are gaining ground in the manufacturing of automotive parts, including gas- and water-assisted injection molding, bright surface molding and also reactive technologies. Reactive injection molding (RIM) and resin transfer molding (RTM), however, cannot be integrated into car factory assembly lines due to their considerably longer cycle times. Despite this, more and more and larger and larger parts are manufactured with these technologies, for example, the whole body of the BMW i3, which is produced with a cycle time of a few minutes [5]. The technology can produce a functional part as a whole composite structure, but recycling is a problem because these technologies produce cross-linked structures. For this reason, much research has been done in the past few years into the practical application of in situ polymerization. The technology produces polyamide structures of a thermoplastic matrix [6]. The advantage of the technology is that the mold is filled with a low-viscosity oligomer, which can also impregnate complex textile systems, and then polymerization also takes place in the mold [7].

**Figure 1:**
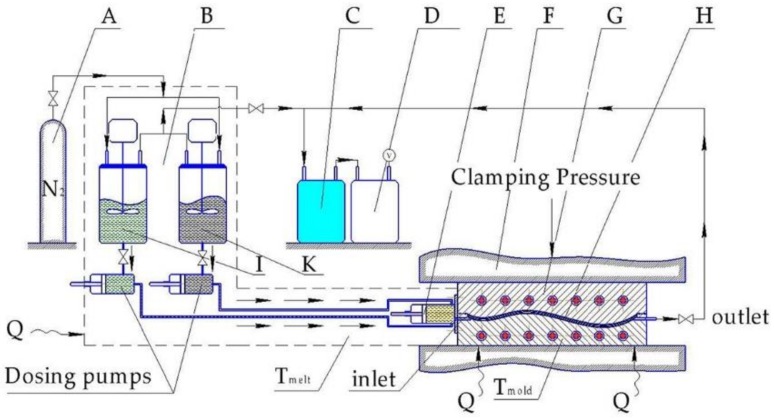
Scheme of the T-RTM process (A—nitrogen source; B—dosing unit; C—cold trap; D—vacuum pump; E—dynamic mixing head; F—mold carrier; G—metal mold; H—textile preform; I—tank with ε-caprolactam + initiator; K—tank with ε-caprolactam + activator [8].

This technology is known as thermoplastic-resin transfer molding (T-RTM), which combines low viscosity to form the complex reinforcement-based structures and the recyclability of thermoplastics. The low-viscosity reactive caprolactam system used in the T-RTM technology completely wets the reinforcement, then polymerizes in the temperature-controlled mold in a few minutes, creating the polyamide 6 polymer. This technology makes it possible to manufacture composite parts at low injection pressure, and with cycle times as short as 1–2 min. The first step of manufacturing is cutting the required geometry from the reinforcement. The layers are placed on top of each other with a binder and preformed in a press. The type of binder has to be chosen so that it does not impair the adhesion between the fibers and the matrix. The preformed parts are cut around their contour and placed in the T-RTM mold. The preheated material reaches the heated mold through a mixing head (Figure 1).

Some of the first industrial parts for the T-RTM technology were produced by injection molding machine developers, such as KraussMaffei [5,7] and Engel [9]. KraussMaffei produced some demonstration parts for the automotive industry, such as a hybrid fiber-reinforced car roof cover frame and a B-pillar [5,7]. They reached 58 to 70 percent fiber content with a cycle time of 2 to 5 min. This demonstration project also involved a compression phase in the process, when the filling phase was finished. With this uniform holding phase, their goal was to reduce shrinkage and void content, and also to improve surface quality. Wakeman et al. [10] also used the advantage of this compression method, where a small gap was maintained in the mold to provide space for injecting extra melt for compensation. 

The edge of products produced by T-RTM have to be either cut off or molded around, due to the characteristics of the technology. The products are typically flat, with a simple geometry, and their required rigidity can be achieved by overmolding ribs on them. As a result, overmolding will be especially important as a supplementary technology of T-RTM. The thermoplastic-based continuous fiber-reinforced preforms and overmolding on them are spreading because of the low mass of the hybrid structure, short cycle times, and because the process can be automated very well and recycled materials can be used. Interfacial adhesion is determined by the temperature of the welding zone, the pressure in the mold cavity, and the intensity of the cooling of the welding zone [11,12,13]. 

Multi-component hybrid thermoplastic parts can have overmolded parts all over the product or overmolded parts, for example ribs, in just some parts. To examine the mechanical properties of the two different parts, Joppich et al. [11] developed two different injection molds. The specimens that they produced were suitable for pure tensile, pure shear and peeling tests. They performed the tensile and peeling tests using a clamping system developed by Weidenmann et al. [14]. The results of the tensile tests had high standard deviation; they explained this by claiming that local defects greatly influence the measurement results, and also when the samples were prepared, some elements may have been weakened during cutting, and perhaps in the relatively small welding areas there may have been differences in the morphology of the preform. They claimed that grouping the samples according to the flow path would have decreased the standard deviation of the measurement results, but later—as opposed to this—they pointed out that they could not differentiate the samples right after the gate or at the end of the flow. The variance of the pure shear test was far lower, which indicates good reproducibility, and suggests that due to the larger connecting surface, local defects affect the shear strength of the structure to a lesser extent. They performed the peeling test of the overmolded rib according to the DIN EN 6033 standard. The results had little standard deviation, which shows good reproducibility, and proves that the method can be used in the testing of overmolding.

Kisslinger et al. [15] designed an injection mold which had an insert that could be moved when the mold was closed, and had it manufactured. With this mold, they produced specimens with which they examined the bonding strength between various materials. After the first component was injected and solidified, the inserts were pulled back while the mold was still closed, and in the space thus freed up, the second component was injected, whose melt front reached the cooled surface of the first component. The authors claimed that the interphase of overmolded parts can be characterized by Computed Tomography (CT) and Raman spectroscopy effectively, which they performed, but they came to their conclusions after testing only one pair of materials with each method without trying other methods. 

Giusti and Lucchetta [16] worked out a method to determine the bonding strength between the components of in-mold forming (IMF) hybrid composites. The matrix of the specimen they made for the tensile tests was PA6 and its preform was a composite plate with fabric as reinforcement. The overmolded component was 50% long fiber PA66. However, their preform was not large enough. They examined seven specimens with each setting at a crosshead speed of 50 mm/min, but before the test, they discarded visibly delaminated specimens, which was a mistake because they did not explain the cause of the defect and did not specify how many defective specimens they had at each setting. They showed with a one-way analysis of variance that both melt temperature and injection rate have a considerable effect on welding strength. 

## 2. Materials and Methods

**Figure 2:**
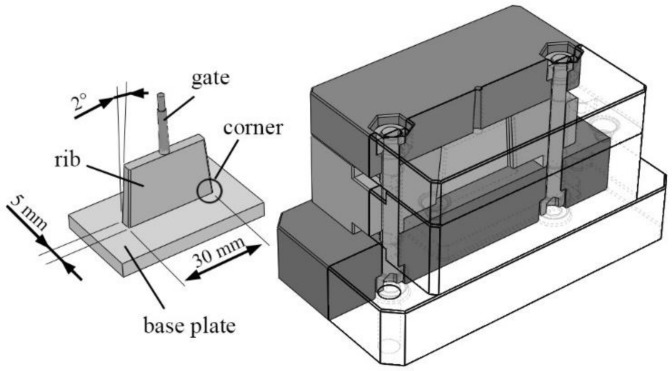
The mold and the part: left—molding (base plate overmolded with a rib through the gate), right—the 3-plate-like aluminum prototype mold (half transparent).

Our goal was to analyze the overmolding of ribs on the PA6 preforms made by in situ polymerization. For this, we designed our own product geometry (Figure 2), in which a rib of a basic area of 30 mm × 5 mm and a height of 15 mm with a side draft of 2° was overmolded on a 50 mm × 30 mm, 2 mm thick base plate. We designed a manually operated prototype aluminum mold for the task (Figure 2) in which the rib was formed with a direct sprue.

We examined preforms of three different materials to obtain information about the effect of the material and the manufacturing technology of the preform on the bond strength of the rib. The first preform was a large sheet of 2 mm thickness, made by magnesium catalyst-based in situ polymerization (IS-A). The second material was the Brüggemann AP-NYLON-based (Versmold, Germany) in situ polymerization plate where 3% of Brüggemann Brüggolen C10 was used as catalyst and 3% of Brüggemann Brüggolen C20P was used as activator (IS-B). The third preform was an injection-molded PA6 (Durethan B30S, Lanxess GmbH, Cologne, Germany) plate of 80 mm × 80 mm and its thickness was also 2 mm (IM). 

The material of the overmolded ribs was also PA6 (Durethan B30S, Lanxess GmbH). The granules—and later on the pre-plates as well—were first dried in a Heraeus UT20 hot-air dryer (Heraeus Holding GmbH, Hanau, Germany) at 80 °C for 6 h. The pellets were injection-molded with an Arburg Allrounder 370S 700-250 injection molding machine (Lossburg, Germany) equipped with a 30 mm diameter, L/D = 25 screw.

**Figure 3:**
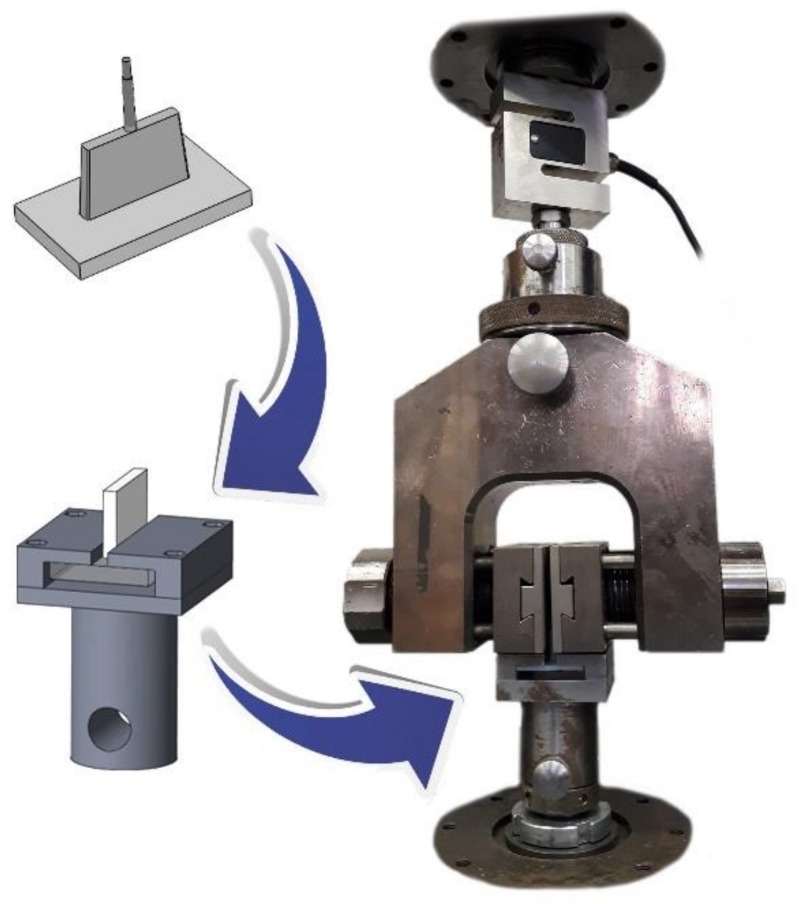
The special grip for the rib pull-off tests.

The tensile tests were performed on a Zwick Z005 universal testing machine (Zwick Roell AG, Ulm, Germany) equipped with a special grip that we designed (Figure 3) to test the debonding force of the ribs. All tests were executed at room temperature and at a relative humidity of 50%. Five specimens were tested for each measurement and a 5 mm/min travel speed was used for the experiments.

## 3. Results and Discussion

For overmolding, the most important parameters are pressure (filling pressure too, but mainly holding pressure) which holds the plate and the rib together, the duration of the pressure (holding time) and also melt temperature and the preheating temperature of the plate. Based on Differential Scanning Calorimetry measurements (ISO 11357-3:2018), the melting temperatures of the different types of PA6 are 218–223 °C, thus the surface temperature of the pre-plate must reach its melting temperature to have a welding effect. The suggested melt temperature range for injection molding varies from 260 °C to 280 °C, therefore the two limits were tested. The suggested mold temperature range varies from 80 °C to 100 °C, but the mold temperature for the prototype mold cannot be controlled precisely, thus it was controlled externally between the cycles, then the temperature was measured with a thermal imaging camera right after the cycle. Three different mold temperatures were used; the low mold temperature was about 30 ± 10 °C, the medium temperature was 50 ± 10 °C, while the high temperature was 70 ± 10 °C.

**Figure 4:**
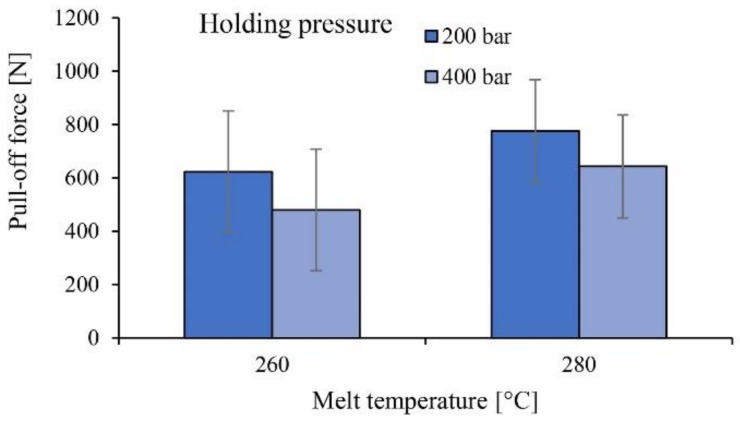
The pull-off force with the low and the high melt temperature.

With the first tests the effect of the melt temperature of the injected material was evaluated. The base plates were the commercially available plates produced by in situ polymerization. The mold was cold; all the plates were kept cold for the measurements and prior to injection molding, their surface was cleaned with acetone. As expected, the pull-off force increased with melt temperature (Figure 4).

**Figure 5:**
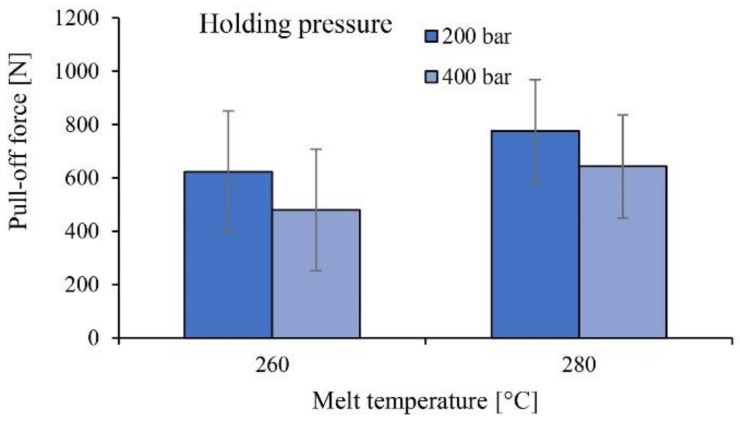
The pull-off force as a function of holding time.

The holding pressure was set to 200 bar and 400 bar for two series of tests, while holding time was set to 15 s. Contrary to expectations, increasing holding pressure and holding time decreased the bonding strength between the plate and the rib (Figure 5). Welding only occurred in the corners, while the center of the plates only had adhesion. 

**Figure 6:**
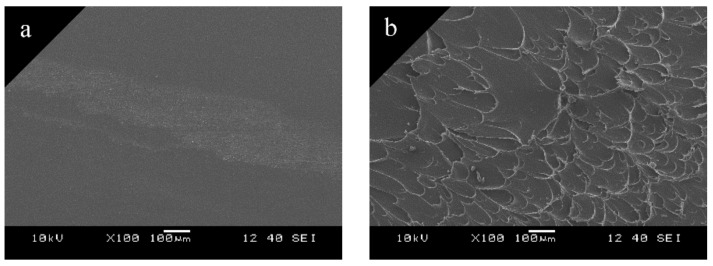
Scanning electron microscopy tests (100 × magnification): (**a**) non-welded; (**b**) welded areas.

We performed scanning electron microscopy tests and found two different surfaces where the base plate and the rib connected. One type was where the overmolded material did not melt the base plate (Figure 6a), while in the other case the overmolded rib melted the base plate (Figure 6b). Figure 6a shows an area at the edge of the middle of the rib. The whitish part is the contour of the edge of the rib, the part above it is the part connected to the rib, while the part below it was covered by the mold and did not come into contact with the melt. These two parts are identical, which proves that in these areas connected to the rib the base plate did not melt and so no cohesion occurred between them. In the case of the other type (Figure 6b) there is a tough break area, which proves that the base plate melted, and cohesion occurred between the two components. In the individual tests, the proportion of these two areas, that is, the melted and non-melted areas, changed.

**Figure 7:**
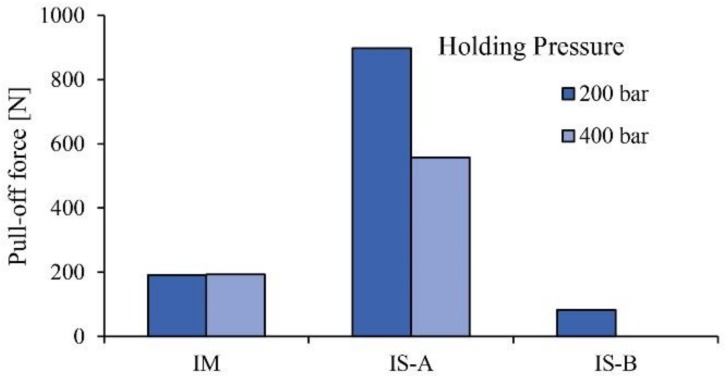
The pull-off force for the three different plates (IM—injection-molded plates; IS-A—magnesium catalyst-based in situ polymerization plates; IS-B—Brüggemann AP-NYLON-based in situ polymerization plates).

Based on these results, for the comparison of the materials, the high melt temperature and the low holding pressure with a short holding time were used. Three different base plates were tested; the reference plate was the commercially available plate produced by in situ polymerization, while the two others were the injection-molded plate and the in situ polymerization-based plate that we produced. As can be seen, the best results were achieved with the in situ polymerization-based plates, while the two others showed greatly inferior results (Figure 7).

The results are in parallel with the residual monomer content of the given materials. It, however, cannot be proved that this is the root course of the welding problems. We used thermogravimetric analysis (TGA, Q500, TA Instruments, New Castle, DE, USA) to measure the monomer content of the plates at 200 °C and the lowest monomer content was found in the commercially available in situ polymerization plate, which was 1.34%. The next was the injection-molded material, which had 1.49% of monomer content, while the worst results came from the test version of the in situ polymerization material, where the residual monomer content reached 1.82%.

**Figure 8:**
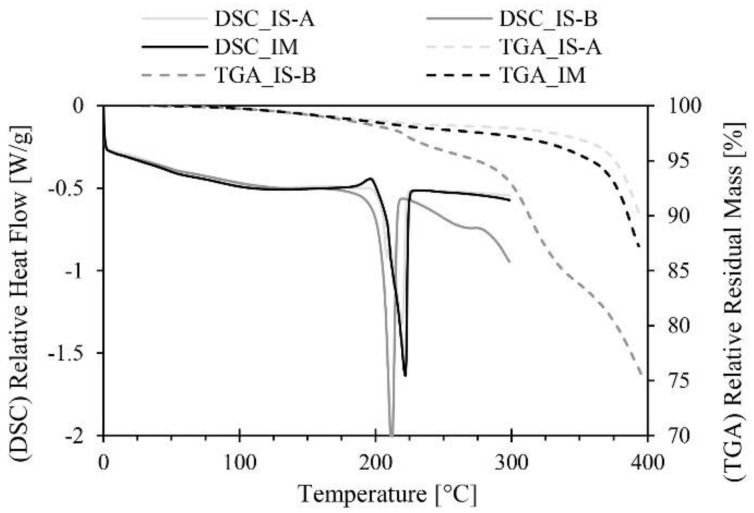
Differential scanning calorimetry (DSC) and thermogravimetric analysis (TGA) curves for the three different plates.

As can be seen in Figure 7, the mechanical properties of the IS-B material (Brüggemann AP-NYLON-based in situ polymerization plate with 3% catalyst and 3% activator) are extremely weak. This phenomenon is a consequence of the degradation (depolymerization) of the material over its melting temperature, as can be seen in Figure 8 in the TGA results. Contrary to the two consumer materials (IS-A, IM), the material we produced does not contain a stabilizer, thus significant degradation occurred over its melting temperature.

**Figure 9:**
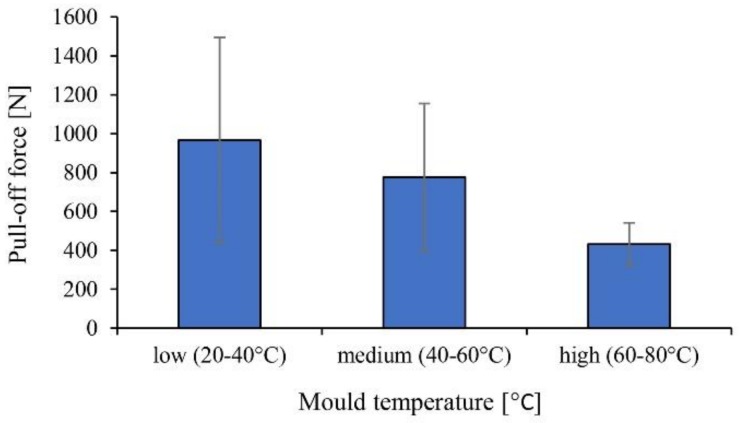
The pull-off force as a function of mold temperature (commercially available in situ PA6 plate (IS-A); holding pressure was 200 bar and holding time was 1 s).

Although we used an aluminum prototype mold without a cooling system, the approximate mold temperature was controlled externally between the cycles, then the temperature was measured with a thermal imaging camera right after the cycle. The low mold temperature was about 20–40 °C, the medium temperature was 40–60 °C, while the high temperature was 60–80 °C. With increasing mold temperature, the possible monomer content of the surface of the plates increase, thus the pull-off forces decrease accordingly. Also, standard deviation decreases as mold temperature increases because the possible monomer content on the surface more uniformly decreases the binding force (Figure 9).

**Figure 10:**
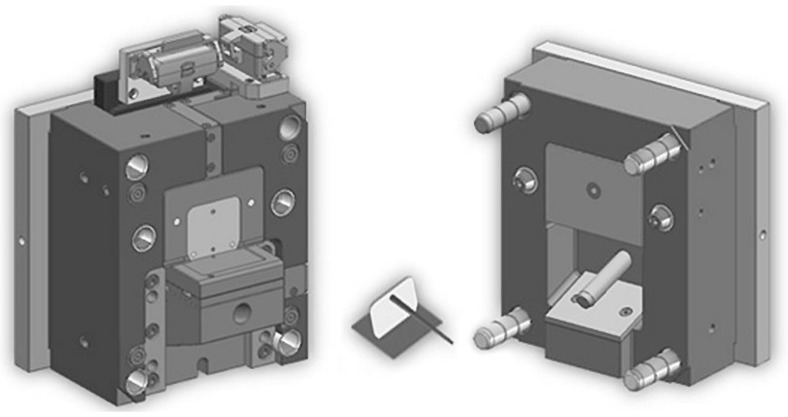
Injection mold for the overmolding investigation equipped with pressure and infrared temperature sensors.

To verify the results obtained in this paper, we designed a mold suitable for overmolding, which can overmold a 70 mm × 50 mm rib on an 80 mm × 80 mm preform (Figure 10). The mold was designed to incorporate two pressure sensors and three infrared (IR) temperature sensors for the analysis of the welding environment. The temperature of the welding environment can be analyzed with the help of the temperature sensors, both from the side of the preform and the side of the rib, close to the actual surface of welding. Also, temperature control of the mold and the inserts were designed so that the temperature of the preform could be controlled well and the temperature could be set over 100 °C as well. 

## 4. Summary

Our goal was to investigate the reinforcement of products manufactured by in situ polymerization with injection-molded ribs. To this end, we developed a prototype product and mold which made it possible to overmold on different preforms with different technological parameter settings. We used three different materials for the plate preforms. The first preform was from a material made by in situ polymerization and with a magnesium catalyst. The second material was a Brüggemann AP-NYLON-based in situ polymerization plate. The third preform was an injection-molded PA6 (Durethan B30S, Lanxess GmbH) plate. 

Through the pull-off test of the overmolded ribs, we pointed out that in most cases, most technological settings produce an effect opposite to what is expected. The only exception was melting temperature; increasing melt temperature improved binding strength between the rib and the plate. On the other hand, increasing holding pressure and holding time impaired the quality of welding. We assume that there is a connection between monomer migration and binding strength in the case of the individual materials. Thermogravimetric analysis (TGA) showed that the most stable material is the Polyamide (PA) made by in situ polymerization and with a magnesium catalyst—it showed the best welding properties in the investigated range of technological parameters. Increasing mold temperature also impaired the efficiency of binding, which is presumably also connected to the depolymerization processes of the base material. 

We designed an injection mold suitable for producing overmolded parts. We are going to investigate the effect of injection molding parameters on the binding strength between the preform and the overmolded part with the help of pressure and temperature sensors designed to be in the mold.
